# Statistical and clustering validation analysis of primary students' learning outcomes and self-awareness of information and technical online security problems at a post-pandemic time

**DOI:** 10.1007/s10639-022-11436-3

**Published:** 2022-11-17

**Authors:** Taras Panskyi, Ewa Korzeniewska

**Affiliations:** 1 Institute of Applied Computer Science, Lodz University of Technology, Stefanowskiego 18/22, 90-537 Lodz, Poland; 2 Institute of Electrical Engineering Systems, Lodz University of Technology, Stefanowskiego 18/22, 90-924 Lodz, Poland

**Keywords:** Primary school education, Online security, e-threats, Cyberspace, Self-awareness

## Abstract

The authors decided to investigate the impact of the pandemic period and the resulting limitations in Polish primary school online security education. The first part of the study investigates the impact of the COVID-19 pandemic on students’ educational learning outcomes in information and Internet security. The study has been performed via a student-oriented survey of 20 questions. The statistical analysis confirms the significant difference before and after the pandemic in several questions at most. Nevertheless, this justifies the statement that pandemics had a positive impact on post-pandemic Internet-related security education. The second part of the study has been focused on students' perception and self-awareness of cyberspace problems. For this purpose, the authors used novel majority-based decision fusion clustering validation methods. The revealed results illustrate the positive tendency toward the students' self-awareness and self-confidence of online security problems and e-threats before, during and after the challenging pandemic period. Moreover, the presented validation methods show the appealing performance in educational data analysis, and therefore, the authors recommended these methods as a preprocessing step that helps to explore the intrinsic data structures or students' behaviors and as a postprocessing step to predict learning outcomes in different educational environments.

## Introduction

COVID-19 has significantly reshaped not only the way of social life but also the way of delivering the proper education. Virtual interactions became increasingly favored over in-person meetings, and private video chat sessions rapidly replaced public gatherings. Crisis-prompted education with distance and/or hybrid learning was strongly recommended and implemented, school classes were frequently conducted online while seminars and exams migrated to webinars. Outdoor sporting activities were also reduced while home/indoor activities increased, with ICTs and the Internet enabling many processes. Moreover, even STEM school education that includes laboratory classes as practical components to an otherwise theoretical material has been transferred into the virtual domain without significant losses in educational learning outcomes (Panskyi et al., 2021a).

Due to the pandemic, people started to spend more time on social media tools, shopped online, watched movies, and played video games more than usual by connecting to the Internet (King et al., 2020; Nielsen Global Media, 2020). Digital networks that deliver the Internet and the services that ride on those networks have leapt from an ancillary “nice to have” to something that is critical and crucial to social, economic, educational and personal development in our daily lives. Nowadays in the fast-changing post-pandemic digital world, technology-led learning is becoming the norm and students may be using ICTs before they can read and write. Students use their computers and laptops during distance classes, mobile phones to keep in contact with friends or parents and tablets for entertainment. With the closure of many schools, due to the pandemic, students have been forced to study and communicate only via the Internet (Dong et al., 2020). The Internet has become an essential tool for all students around the world. During the pandemic, while restrictions were in place, and social and economic activities become more digital, students, parents and teachers could rely on the Internet and connectivity. Thanks to broadband networks and digital infrastructure, students could keep on learning, socializing and working. Nowadays, no one can deny the significant Internet’s role in keeping education, personal lives and businesses amid the global post-pandemic crisis.

New ICT technologies and Internet-related services allow not only faster and easier access to information, cheaper services or interesting forms of entertainment, but also are known for their "dark" side; using ICTs can be risky, especially for primary school students, that can easily lose control over information about themselves and their life on the Internet (Fundacja Panoptykon, 2015). In the post-pandemic era parents, teachers and students struggle with the new sophisticated problems posed by the global network. One of the problems related to the availability of modern ICTs as well as the frequency of using the global network and the time spent on the Internet is the problem of e-addiction. Internet addiction is a broad term that covers a range of behaviors and impulse-control problems involving the Internet, personal computer, and mobile technology (Grzega, 2021). Warning signs of Internet addiction include regular network consumption at the expense of other interests, signs of aggression if access to the Internet global network proves impossible and not caring about relations with one’s family or friends (Kisiel & Woźnialis, 2021). Other problems also could be associated with conditions or students' behaviors that involve excessive or problematic use of the Internet, namely, an unprecedented rise in screen time, self-efficiency, mental health and crisis, hikikomori (Ferrara et al., 2020) coping with stress, digital fatigue, grooming (Pisarska & Ostaszewski, 2020; Saggers, 2020), gambling (Donati et al., 2021; Gjoneska et al., 2022; (Serra et al., 2021), cyberbullying (Yang et al., 2022), cyberstalking (Dhillon & Smith, 2017), cyberscams (Whitty, 2020) and numerous other consequences and e-threats of Internet-related post-pandemic education (Tomczyk & Walker, 2021; Lazarinis et al., 2019; Rothe et al., 2021; Zuo et al., 2021).

According to GUS statistics (GUS, 2020a) in 2020, 89.6% of all households in Poland had broadband Internet access. Another GUS report (GUS, 2020b) revealed that more than 91% of households with children had a broadband Internet connection. Moreover, the Fundacja Stocznia (2021) study demonstrated an increased value of almost 99% in 2021. With respect to the ICTs, the MEN study (MEN, 2020) and the Report of the Organisation for Economic Cooperation and Development (OECD, 2020) confirmed that Polish primary students were technologically prepared for the transition to distance education. Based on PISA data (Digital Center, 2020). only 1–1.5% of primary school students in 2021 did not have a computer or tablet at home. Moreover, the authors (Panskyi et al., 2021b) declared that Polish primary school students during the pandemic reveal a high level of ICT access and ICT utilization. With respect to the time spent on the Internet according to CBOS study (CBOS, 2018), students aged 14–16 use their ICT devices approximately 4–5 h a day. Before the pandemic in 2019, almost 15.3% of students spent 1–2 h using their ICTs, 27.3% spent 2–4 h and 22.9% spent 4–6 h a day (NASK, 2019). During the pandemic, at the beginning of 2020, the average time spent by III-VIII grade primary students using was 5 h a day, in May 2020 the average increased to 9 h a day (FEZiP, 2021). Due to the pandemic, the crisis-prompted distance education had forced students to spend significantly more time using their ICT devices than before it starts.

In the pre-pandemic period, students used their ICTs to search for information on the Internet, to communicate with others using social networks or to play computer games. The use of ICTs to participate in distance education classes was very rare. Only a small amount of lessons was conducted with the use of such ICTs. The period of the pandemic and the necessity to communicate with the use of the Internet favored the development of software for remote communication and conducting online classes in an accelerated mode. This resulted in the manifestation of a previously unknown working virtual environment full of dangers and threats related to the security of communication. The pandemic has brought into sharp focus how privileged some subjects are, and how others have been left behind. It has also shown that teachers could succeed, even during a crisis, with the right ICTs, adapted distance curriculum and support system. However, in such an environment, teachers, parents and students did not pay attention to all the digital communication channels they used, permanently attending to and unsafely seeking out information. For this reason, the authors decided to investigate the selected problems of e-threats in primary school settings. Moreover, the Internet and information security problems are discussed in informatics subject according to the new Polish reform in the primary school education initiated in the school year 2017/2018. With respect to the new reform and corresponding Core Curriculum, all children (aged 7–15/16) are required to learn informatics in primary schools (Sysło, 2019, 2020). Therefore, the authors made the attempt to investigate the students’ learning outcomes in the security areas before, during and after the pandemic. Our study is focused only on one basic distinctive kind of e-threat in cyberspace (Zdzikot, 2021) such as malware infection (viruses, worms and trojans), phishing, password management, identity theft, grooming and other aspects of information and technical Internet security (Rowicka, 2018).

In the first part of the article, the authors analyzed the selected security problems the Polish primary school students could face before, during and after the pandemic challenging period. First, a descriptive analysis has been carried out followed by an inferential analysis performed using the well-known statistical methods. The authors decided to analyze the impact of the pandemic period and the resulting limitations to primary school students' preparedness for Internet and information security problems and the capability of proper response to these hazard emergencies.

Thereafter, the students’ perception and self-awareness of the information and Internet security problems before, during and after the pandemic have been also studied. The authors revealed groups (clusters) of security problems that should be outlined, structured and discussed in greater depth. For this purpose, clustering validation methods have been used. Among the variety of validation techniques, the authors offered their own solutions, unique and novel majority-based decision fusion methods (MBDFMs) based on the pretest–posttest strategy (Panskyi & Mosorov, 2021). Despite the rest of the decision fusion techniques, the presented new MBDFMs do not require weighting procedures, the choice of the appropriate clustering algorithm and dissimilarity measure. Moreover, the MBDFMs are reliable enough to cope with multidimensional noisy data with different densities and overlapping clusters. The authors had made an attempt to integrate the MBDFMs for educational data analysis. In contrast to classical statistical methods, the proposed clustering MBDFMs allow to unmask, visualize and illustrate a possible potential change in students' perception and self-awareness of online security problems under the influence of the COVID-19 pandemic. Finally, the authors made a set of recommendations for further research and suggestions for developing the self-awareness of teachers, parents and students addressed to crucial information and Internet security problems.

In these aspects, it became purposeful to pose crucial research questions:

1. Do the post-pandemic Polish primary school students' educational learning outcomes in information and Internet security significantly differ from the pre-pandemic period?
2. Are the majority-based decision fusion solutions suitable, efficient and robust for educational data analysis and informative, illustrative and transparent to answer the question about the influence of the pandemic on the primary students’ perception and self-awareness of e-threats in primary school settings?

## Methodology

### Data collection

For this study, a non-experimental research design has been proposed, during 2019/2020, 2020/2021 and 2021/2022 academic years. The research conducted before the pandemic comes was an auditorium survey. During the pandemic and in the post-pandemic period the research was conducted using the auditorium internet survey. Internet survey methodology is a variation of the CAWI (Computer Assisted Web Interviews) research technique, in which the study is carried out online using a questionnaire placed in a virtual educational environment. A structured questionnaire designed by the Polish Ministry of Administration and Digitization devoted to the annual Safe Internet Day was adapted to the age groups of primary school students (aged 8–10 years) to map their perception, use, and knowledge toward the e-threats. Participation in the study was anonymous. In order for the students to take part in the survey, the written consent of their parents was also required.

The survey included 20 close-ended multiple-choice questions (Q1-Q20) about: technical e-threats: viruses, firewall, malware; information security problems: undertaking online activities, contact with dangerous content on the Internet, data confidentiality; social threats: dangerous contacts, grooming. The questions consist of one true/correct answer and three distractors. The Boolean logic has been used for statistical analysis of each Q1-Q20 question, where the "true" answer is set to be "1" and the false answers to be "0". The list of the questions from the survey is presented in Table 3 (see Appendix 1).

### Participants

The study sample consists of 243 primary school students from K2 to K4 curriculum standards in Poland. The following groups participated in the presented research study:

- Pre-pandemic in 2019/2020 school year: 78 students;
- During pandemic in 2020/2021 school year: 83 students;
- Post-pandemic in 2021/2022 school year: 82 students.

In order to ensure that the results are not influenced by the size of the sample of students three target groups were selected with almost the same number of students. Moreover, the students were assigned to the group “1” – pre-pandemic, “2” – during pandemic “3” – post-pandemic respectively. Each group of students represents an independent set, that is, the students were selected from three different schools in the Lodz Voivodeship in central Poland.

### Descriptive statistics

The descriptive statistics presented in Table 1 include the short names for each dimension, the mean, standard deviation, skewness and kurtosis of the students’ answers. According to Bai and Ng (2005), data are normally distributed when skewness and kurtosis are respectively within the range of—1 and—3. Table 1 shows that the data distribution of all three groups of students was close to a normal distribution.

**Table 1:** The descriptive statistics of the analysed survey regarding the students' educational outcomes in the selected security e-threats, assembled in three distinctive groups (before, during and after the pandemic)

	Pre-pandemic				During pandemic				Post-pandemic			
	M	SD	SK	KU	M	SD	SK	KU	M	SD	SK	KU
Q1	0.46	0.50	0.16	-2.02	0.50	0.50	0.02	-2.05	0.49	0.50	0.05	-2.05
Q2	0.23	0.42	1.30	-0.31	0.25	0.44	1.16	-0.68	0.29	0.46	0.93	-1.17
Q3	0.18	0.39	1.70	0.92	0.14	0.35	2.06	2.29	0.21	0.41	1.47	0.17
Q4	0.58	0.49	-0.31	1.95	0.54	0.50	-0.17	-2.02	0.57	0.50	-0.30	-1.96
Q5	0.47	0.50	0.10	-2.04	0.57	0.50	-0.27	-1.97	0.62	0.49	-0.51	-1.78
Q6	0.52	0.50	-0.10	-2.04	0.58	0.50	-0.32	-1.94	0.61	0.49	-0.46	-1.83
Q7	0.32	0.47	0.78	-1.42	0.40	0.49	0.43	-1.86	0.61	0.49	-0.46	-1.83
Q8	0.26	0.44	1.14	-0.72	0.30	0.46	0.88	-1.25	0.32	0.46	0.80	-1.39
Q9	0.38	0.49	0.48	-1.81	0.41	0.49	0.37	-1.90	0.52	0.50	-0.10	-2.04
Q10	0.67	0.47	-0.72	-1.52	0.72	0.45	-1.01	-0.99	0.76	0.43	-1.21	-0.54
Q11	0.44	0.50	0.26	-1.99	0.61	0.49	-0.47	-1.81	0.57	0.50	-0.30	-1.96
Q12	0.50	0.50	0.00	-2.05	0.54	0.50	-0.17	-2.02	0.59	0.50	-0.35	-1.92
Q13	0.46	0.50	0.16	-2.02	0.43	0.50	0.27	-1.97	0.57	0.50	-0.30	-1.96
Q14	0.41	0.49	0.37	-1.91	0.36	0.48	0.59	-1.69	0.46	0.50	0.15	-2.03
Q15	0.56	0.50	-0.26	-1.98	0.58	0.50	-0.32	-1.94	0.60	0.49	-0.40	-1.88
Q16	0.40	0.49	0.43	-1.86	0.41	0.50	0.37	-1.90	0.67	0.47	-0.74	-1.49
Q17	0.74	0.44	-1.14	-0.72	0.75	0.44	-1.15	-0.68	0.80	0.40	-1.57	0.47
Q18	0.36	0.48	0.60	-1.68	0.66	0.48	-0.70	-1.55	0.72	0.45	-0.99	-1.03
Q19	0.44	0.50	0.26	-1.98	0.54	0.50	-0.17	-2.02	0.73	0.48	-0.57	-1.72
Q20	0.21	0.41	1.49	0.22	0.22	0.42	1.40	-0.04	0.24	0.43	1.21	-0.54

The analysis of the overall survey Q1-Q20 revealed the highest mean score was in question Q17— 0.80 for group „3″ after the pandemic. The lowest mean score of the overall survey was shown in question Q3—0.14 for group „2″ during the pandemic. The pairwise analysis of the mean scores of two groups („1″ and „2″) of the presented survey Q1–Q15 discovered the superiority of group „2″ over group „1″ in all questions except Q3, Q4, Q13 and Q14. With respect to these groups, the question Q18 revealed the biggest difference—0.3 in favor of group „2″ and questions Q16, Q17 and Q20—the smallest difference—0.01 in favor of group „2”.

The pairwise analysis of the mean scores of two groups („2″ and „3″) revealed the superiority of the group „3″ over the group „2″ in all questions except Q1 and Q11. Question Q16 revealed the biggest difference—0.26 in favor of group „3″ and the question Q1—the smallest difference—0.01 in favor of group „2”.

Finally, the pairwise analysis of the mean scores of two groups („1″ and „3″) showed the superiority of the group „3″ over the group „1″ in all questions except Q4. Regarding the analysis, question Q18 revealed the biggest difference—0.36 in favor of group „3″ and the question Q4—the smallest difference—0.01 in favor of group „1”.

With respect to the group, and related pre, during and post-pandemic time, the highest mean score was revealed in question Q17— 0.74 before the pandemic, 0.75 during the pandemic and 0.80 after the pandemic, while the lowest was in question Q3- 0.18, 0.14 and 0.21 before during and after the pandemic respectively. Question Q17 is related to the grooming, sexting and privacy e-threats. Question Q3 is related to the ICTs, viruses and Internet-related technical security problems. At first glance, all three groups had security knowledge gaps in the field of Information security issues, on the other hand, it could be stated that pandemic doesn't significantly influence the student outcomes in the technical security domain. However, the biggest difference between the highest Q17 and the lowest Q3 mean scores have been revealed within the group „2″– 0.61 which can be considered as a negative pandemic influence on students' basic Internet-related security outcomes. However, to gain crucial insights into the differences in students' security knowledge and Internet safety experience before, during and after the pandemic a comprehensive analysis should be performed and the results should be discussed in greater depth.

## Statistical analysis

To verify the significance of the model proposed in relation to the different groups, a multiple comparisons one-way ANOVA has been used. p. < 0.05 was considered statistically significant. All analyses were conducted using the Statistical Package for the Social Sciences (IBMSPSS, version 21). Analysis of internal consistency showed that the overall Cronbach alpha obtained for the Q1–Q20 questions was 0.91. Levene’s homoscedasticity assumption was fulfilled, so multiple comparisons have been made by Turkey. Table 2 shows the results of the ANOVA analysis conducted for all students’ Q1–Q20 answers based on the presented survey.

**Table 2:** Multiple comparisons of primary school students’ informatics learning outcomes in selected Internet and information security problems

	(I) Group	(J) Group	Mean Difference (I-J)	Std. Error	Sig	95% Confidence Interval	
						Lower Bound	Upper Bound
Q1	1	2	-0.032	0.079	0.912	-0.219	0.154
	2	3	0.006	0.078	0.997	-0.178	0.191
	3	1	0.026	0.079	0.942	-0.161	0.214
Q2	1	2	-0.022	0.069	0.945	-0.186	0.141
	2	3	-0.039	0.068	0.832	-0.201	0.122
	3	1	0.061	0.069	0.648	-0.102	0.226
Q3	1	2	0.046	0.059	0.712	-0.094	0.188
	2	3	-0.074	0.059	0.415	-0.214	0.064
	3	1	0.027	0.059	0.888	-0.113	0.169
Q4	1	2	0.034	0.078	0.898	-0.150	0.220
	2	3	-0.031	0.077	0.916	-0.214	0.152
	3	1	-0.003	0.078	0.999	-0.189	0.182
Q5	1	2	-0.131	0.078	0.215	-0.315	0.053
	2	3	-0.067	0.077	0.654	-0.249	0.114
	3	1	0.198	0.078	0.031*	0.014	0.383
Q6	1	2	-0.052	0.078	0.780	-0.237	0.132
	2	3	-0.031	0.077	0.913	-0.213	0.150
	3	1	0.084	0.078	0.533	-0.101	0.269
Q7	1	2	-0.077	0.076	0.572	-0.257	0.103
	2	3	-0.212	0.075	0.015*	-0.390	-0.034
	3	1	0.289	0.076	0.001*	0.108	0.470
Q8	1	2	-0.044	0.072	0.808	-0.214	0.125
	2	3	-0.015	0.071	0.973	-0.183	0.151
	3	1	0.060	0.072	0.679	-0.109	0.231
Q9	1	2	-0.025	0.078	0.945	-0.209	0.159
	2	3	-0.114	0.077	0.299	-0.296	0.067
	3	1	0.139	0.078	0.178	-0.045	0.324
Q10	1	2	-0.056	0.071	0.711	-0.224	0.112
	2	3	-0.033	0.070	0.885	-0.199	0.132
	3	1	0.089	0.071	0.425	-0.079	0.258
Q11	1	2	-0.178	0.078	0.060	-0.362	0.005
	2	3	0.041	0.077	0.854	-0.140	0.223
	3	1	0.137	0.078	0.188	-0.047	0.322
Q12	1	2	-0.042	0.078	0.854	-0.228	0.143
	2	3	-0.043	0.077	0.844	-0.226	0.140
	3	1	0.085	0.079	0.528	-0.101	0.271
Q13	1	2	0.027	0.078	0.934	-0.157	0.213
	2	3	-0.139	0.077	0.174	-0.322	0.043
	3	1	0.111	0.078	0.335	-0.074	0.297
Q14	1	2	0.048	0.077	0.805	-0.134	0.232
	2	3	-0.101	0.076	0.381	-0.283	0.079
	3	1	0.053	0.078	0.775	-0.130	0.237
Q15	1	2	-0.014	0.078	0.982	-0.198	0.170
	2	3	-0.019	0.077	0.966	-0.201	0.163
	3	1	0.033	0.078	0.905	-0.151	0.218
Q16	1	2	-0.012	0.076	0.986	-0.193	0.168
	2	3	-0.261	0.075	0.002*	-0.439	-0.082
	3	1	0.273	0.076	0.001*	0.091	0.454
Q17	1	2	-0.003	0.067	0.999	-0.161	0.154
	2	3	-0.057	0.066	0.657	-0.214	0.098
	3	1	0.061	0.067	0.634	-0.097	0.220
Q18	1	2	-0.303	0.074	0.000*	-0.478	-0.128
	2	3	-0.056	0.073	0.718	-0.229	0.115
	3	1	0.360	0.074	0.000*	0.185	0.535
Q19	1	2	-0.106	0.078	0.363	-0.290	0.077
	2	3	-0.091	0.077	0.458	-0.273	0.089
	3	1	0.198	0.078	0.032*	0.013	0.382
Q20	1	2	-0.011	0.065	0.983	-0.167	0.143
	2	3	-0.027	0.065	0.909	-0.180	0.126
	3	1	0.038	0.066	0.828	-0.117	0.194

* The main difference is significant at the 0.05 level

Table 2 shows the statistically significant differences in question Q5 between group “1” and group “3” (p. = 0.031) and (p. = 0.001). Moreover, the differences in question Q7 have been revealed also between group “2” and group “3” (p. = 0.015) These differences indicate how a pandemic could fundamentally boost the students’ learning outcomes towards the technical e-threats, in particular viruses. The statistically significant differences also have been revealed in questions Q16, Q18 and Q19. Question Q16 portrays the differences between groups “2” and “3” (p. = 0.002) and groups “1” and “3” (p. = 0.001). The following question Q18 indicated the significant differences between groups “1” and “2” (p. = 0.000) and groups “1” and “3” (p. = 0.000). Last but not least, question Q19 demonstrates the differences between group “1” and group “3” (p. = 0.032). Questions Q16, Q18 and Q19 relate to the same category of information security problems in particular the identity theft (personal data such as a name, surname, an identification number, location data, an inter-net identifier, personal image, etc.), grooming and sexting attempts with possible subsequent offences against privacy and hunting for personal data.

Research conducted in 2017 revealed that 83% of students aged 10 to 13 are aware of the dangers and risks of using the Internet (IPC, 2017). Moreover, almost 19.2% of students knew only about some of the technical security issues in particular they define the awareness of hacker attacks and viruses; 11% of students were aware that they cannot be sure who they are talking to on the Internet. Another research conducted in 2018 revealed that almost 19% of primary school students during the year had viruses or other malware on their ICTs (Pyżalski et al., 2019). One-fourth of young people admitted that they had met an adult they met on the Internet: 39% of them informed their parents about it, and 29%—no one (NASK, 2016). More than half of young people – 58%, noticed the presence of the phenomenon of sending erotic photos among their peers (Wójcik & Makaruk, 2014). This is also confirmed by another study (NASK, 2014), according to which 25% of young Internet users admitted that they had received sexting materials. Moreover, 42% of young people aged 15–18 have received a nude photo or video from another person on the Internet (NASK, 2017). Every twentieth child (5%) was persuaded to engage in sexual behavior by a person they met on the Internet. During the pandemic in 2020, the Dyżurnet.pl one of the NASK divisions responded to reports received from Internet users about potentially illegal material (mainly related to the sexual abuse of children and child pornography) with almost 2 600 notifications about child sexual abuse and about 880 dedicated to child sexual exploitation (NASK, 2020). Only 11% of young people regularly change their password to access e-mail accounts and social networks, 13% have experienced identity theft, 14% frequently share online their confidential data and 40% have heard that their friends have experienced such a practice (Siemieniecka et al., 2020). According to numerous studies, risky behaviors most often co-exist with each other. Primary school students undertake risky activities not only online, however, it also confirms their vulnerability to online dangers (Bochenek et al., 2018).

Many studies have diagnosed the problem of the frequent lack of support in developing digital competencies on the part of parents. The analysis shows that although parents admit that they are responsible for the safety of the youngest people online (TNS Polska S.A., 2016), children do not perceive them as guides in the virtual world (Piecuch, 2017). About 60% of parents, who were asked about the reasons for sharing mobile devices with their preschool children, declared that they keep their children entertained in this way (Bąk, 2015). The aforementioned research from 2017 showed that in Poland, most parents do not use any security measures on ICT devices to which their children have access. The report also found that parents rarely talk to children about online safety and information security (Makaruk et al., 2017). The existing knowledge about effective protection against e-threats indicates the enormous role of parents, the emotional climate of young people in family relationships, as well as in the school and peer environment (Walęcka-Matyja, 2013). Nevertheless, the topic of Internet security is still insufficiently present in Polish households (TNS Polska S.A., 2016). It turns out that as much as 23% of parents did not talk to their children about the Internet and information security at all (Makaruk et al., 2019).

Teachers are also responsible for the safe and effective use of ICTs by primary school children (Andrzejewska, 2014; Ciemcioch, 2017). An indispensable element of effective preventive measures is the development of relevant digital competencies of the teaching and pedagogical staff in the field of methods and ways of safe using the Internet by young people (Raczykowska, 2019). The pandemic exposed huge gaps in teachers' STEM and informatics knowledge and emphasized their low digital competencies. As shown by various studies conducted during the pandemic, a significant part of the teaching staff complained about problems related to assessing students' outcomes and the transfer of practical skills in the distance learning formula (Plebańska, 2020; Amielańczyk et al., 2020; Biernat, 2020; Sobiesiak-Penszko & Pazderski, 2020). Other issues were related to the development and implementation of distance learning methods (Jaskulska & Jankowiak, 2020). The teachers “thrown into the deep” felt lonely and deprived of help, were overloaded with programs, and flooded with new teaching methods and time-consuming preparation for distance teaching (Plebańska et al., 2020). Nevertheless, the vast majority of the surveyed teachers 93.9% agreed with the statement that distance education during the COVID-19 pandemic allowed them to learn new pedagogic methodology, ICT-based didactic methods and tools, and lesson plans enabling them the effective implementation of acquired knowledge and skills (Fila et al., 2020). Due to all the problems associated with proper implementation of distance education tasks, teachers did not pay appropriate attention to the cyberspace security issues that undoubtedly should move from the margins to the center of the Polish education system.

The duty of schools towards the security issues in the pandemic age was to synchronise the activities of people belonging to various educational groups (teachers, parents, students), implement the appropriate technical and organisational measures, collect the information on e-threats and vulnerabilities and use preventive and admonitory measures to limit their impact on the security of the ICT system, to contact with authorities competent for cybersecurity, to conduct educational activities towards security awareness and finally, to create a “cyberspace security umbrella”, to protect those who fall within the impact of the crisis-prompted pandemic education. Nevertheless, the schools themselves have become a target for cybercrime attacks. According to the CERT Polska 2020 report (CERT/NASK, 2020), among 2873 educational institution websites with the Joomla system, a total of 5175 vulnerabilities were found. Among the 6602 websites based on CMS WordPress, a total of 9210 vulnerabilities had been revealed. Almost 95% of school websites did not have properly configured security mechanisms. At the beginning of May 2019, 663 schools reported that they had received information via e-mail about the planting of an explosive device on the school premises. The attacks were coordinated via an anonymous picture forum (CERT/NASK, 2019). In September 2020, there were from 800 to 950 cyber-attack attempts a week per school, while in July 2021 there were almost 3000. According to Check Point Research, email was the main factor responsible for 72% of the cyber-attacks in Polish schools (Check Point, 2021). In more than two-thirds of e-mail attacks, malicious files contain the.doc and.exe extension – 16% almost 6% were.xlsx documents.

Counteracting the information and technical Internet security phenomena requires a common position of many entities, both policymakers, headmasters, parents/ legal guardians and teachers (NASK, 2022). All these people should take preventive measures to cover the ICT system with the process of risk management for the security of essential services, implement safeguards in the ICT system to control the exchange of web services with these communication and information systems and introduce the multi-level protection of ICT system. The authorities are required to ensure that the schools are adequately equipped in terms of both technical and organisational capabilities to ensure the efficient prevention, detection and response, of ICT systems' incidents and security risks. Moreover, the operational and exploratory control should allow the establishment of quick, purposeful, and necessary intervention in the event of suspicion that the ICT system (school or home computers, phones, tablets connected by a network along with different websites, social networks, emails, forums, blogs, search engines, multimedia streaming services) is infected with dangers in cyberspace. Finally, the authorities should perform the consultative and advisory activities responsible for initiating, coordinating and managing the risks posed to the security of the network and ICT system. The primary role is to disseminate knowledge on security and spread public awareness in this area. This generates demand for various types of educational assistance services, programs, campaigns, projects and tools which are expected to contribute to raising knowledge and cyber awareness in school educational circles.

Teaching primary school students and acquiring the necessary Internet both information and technical Internet security skills are long-term processes, and this needs to be taken into consideration when designing and implementing any cybersecurity mechanisms and requirements. Poland needs to become actively involved, also by allocating sufficient funds for this purpose in the budget, for building professional education and training. At present, it is still difficult to assess the activities of both teachers, students, schools and parents. The creation of the secure ICT educational system and the implementation of solutions and recommendations are ongoing, so it takes time to make a substantive assessment of the activities of the discussed entities. Nevertheless, the present study shows a comprehensive evaluation of students' learning outcomes in information and technical Internet security problems. This justifies the statement that the pandemic had a positive impact on post-pandemic Internet-related security education. E-threat issues have never been considered more important than it is now in the post-pandemic era.

## Cluster analysis

Other important goals are to raise awareness and promote good practices so that students can protect their information better, and to create a safe information and education space to function. These objectives clearly show that cyberspace needs to be considered in terms of challenges and hazards. The government should take measures to raise the students' awareness of e-threats through conducting informational activities on good practices, educational programmes, campaigns, and training, to increase knowledge and build awareness of cybersecurity, including the safe use of the Internet in particular and the cyberspace overall. During the pandemic Polish governmental and non-governmental public and private organisations disseminated knowledge to raise awareness and social expertise related to information security and conducted crucial awareness-building activities in the sphere of online security.

Polish Safer Internet Center (PSIC) is run by the Empowering Children Foundation (Fundacja Dajemy Dzieciom Siłę – FDDS) and by NASK Institute, during the pandemic, implemented three projects: Saferinternet.pl—awareness-raising activities aimed at promoting safer use of the Internet and new ICTs by children and young people; helplines: support for young internet users; Dyżurnet.pl—Hotline receiving reports about illegal internet content, child sexual abuse images and racism. There were some awareness-raising campaigns: “Home Screen Rules”—its aimed to make parents aware of the problem of abuse of screen ICT devices by themselves; “Take care of children’s brains”—the campaign aimed to draw parents’ attention to the need of balance in the use of screen ICT devices by children; hotline campaign against sextortion—devoted to the problem of producing and distributing self-explicit materials by children. Moreover, some educational projects and tools had been created: “Puzzles of Mr. File and Mr. Folder – a web application for teachers”, “Mr. File and Mr. Folder – on paths of the internet”, „Keeping Children and Young People Safe Online”, “YouTube World”, “What is my child doing on YouTube?”, Necio.pl, Sieciaki.pl, Digital Youth project, SELMA project, IMPACT programme (NASK, 2022). Teachers also had the opportunity to increase their awareness and knowledge by taking part in educational training and events: International Conferences “Keeping Children and Young People Safe Online”, conference “Opportunities, challenges, threats—an introduction to the issue of online safety of children and young people”, Safer Internet Day, etc. The authors consider it appropriate and essential to outline the primary school students' self-awareness of online security problems. It is crucial to reveal and evaluate whether the pandemic significantly changed the students' perception of e-threats. Moreover, the authors made an attempt to draw the overall picture students' perception and self-awareness of online security problems before, during and after the pandemic. Moreover, the authors attempted to draw the overall picture of students' perception and self-awareness of online security problems before, during and after the pandemic. Students' self-awareness transformation process should illustrate the tendency for further actions and recommendations for the measures leading to the introduction of the proper solutions both educational, technical and organisational in the context of online security.

The data was collected by means of a pretest–posttest study based on the survey presented in Table 3 (see Appendix 1). A common situation in the evaluation of students' self-awareness is the researcher's possibility to rely on two waves of data only (i.e., pretest and posttest), which profoundly impacts the choice of the possible statistical analyses to be conducted. Indeed, the evaluation of students' self-awareness on a pretest–posttest design has been usually carried out by using classic statistical tests, such as family-wise ANOVA analyses. However, in the second research question dedicated to the students' self-awareness towards the online security problems, the authors adopted the pretest–posttest procedure that should only serve as the data collection instrument for further analysis. The main part of the analysis has been performed using the clustering validation methods, which could represent a useful methodological tool to have a more realistic and informative representation of analysed data.

Clustering, also known as cluster analysis, has become an important technique in machine learning used to discover the natural grouping of the observed data (Nasraoui & Ben N'Cir, 2019). Clustering is a data mining concept that aims at grouping similar data objects while separating dissimilar ones (Han et al., 2011). Thus, in clustering, the issue is to cluster the data into meaningful groups (clusters) without knowledge about classes and therefore it is sutable technique where a priori information is not available from data set (Hassan et al., 2019). Cluster analysis remains the most relevant and robust tool to gain insight into previously unknown grouping structures and to disclose the intrinsic characteristics of data. Clustering has also found many applications in many fields such as health psychology: improvement of the health care system and prevent illness (Clatworthy et al., 2005); market research: helps to segment the market and to determine target markets (Sarstedt & Mooi, 2019); image segmentation: to detect boarders of objects in an image (Siddiqui & Abid Yahya, 2022); web search: to organize the search results into groups and segregate documents into topics (Aggarwal, 2018); gene expression data: to group genes with similar patterns (Maulik et al., 2011); network: to finding community structures (Mosorov et al., 2015), etc., facilitating the development of hundreds of approaches and thousands of clustering algorithms (Gan et al., 2014). The categorization of clustering methods is neither straightforward nor canonical (Kogan et al., 2006), nevertheless, the major fundamental clustering methods can be classified into the following categories: hierarchical methods, partitioning methods, density-based methods, graph-based methods and grid-based methods.

Applying different clustering algorithms or the same clustering algorithm with varying input parameters on the same given data set can result in diverse partitions and therefore may greatly affect the goodness/quality of clustering results (Mojarad et al., 2019). The effective evaluation of the goodness of clustering results is the main objective of clustering validation. This involves not only finding the “true” number of clusters but also the optimal partition that best fits the underlying grouping structure of the data set. The cluster validity index (CVI) is an indicator, criteria or measure by which to provide a way of validating the quality of clustering algorithms in determining the correct true number of clusters in data sets (Rojas‐Thomas & Santos, 2021). In general, CVIs tailored to quantitatively evaluate clustering results are classified into two groups: internal and external (Halkidi et al., 2001).

**Fig. 1:**
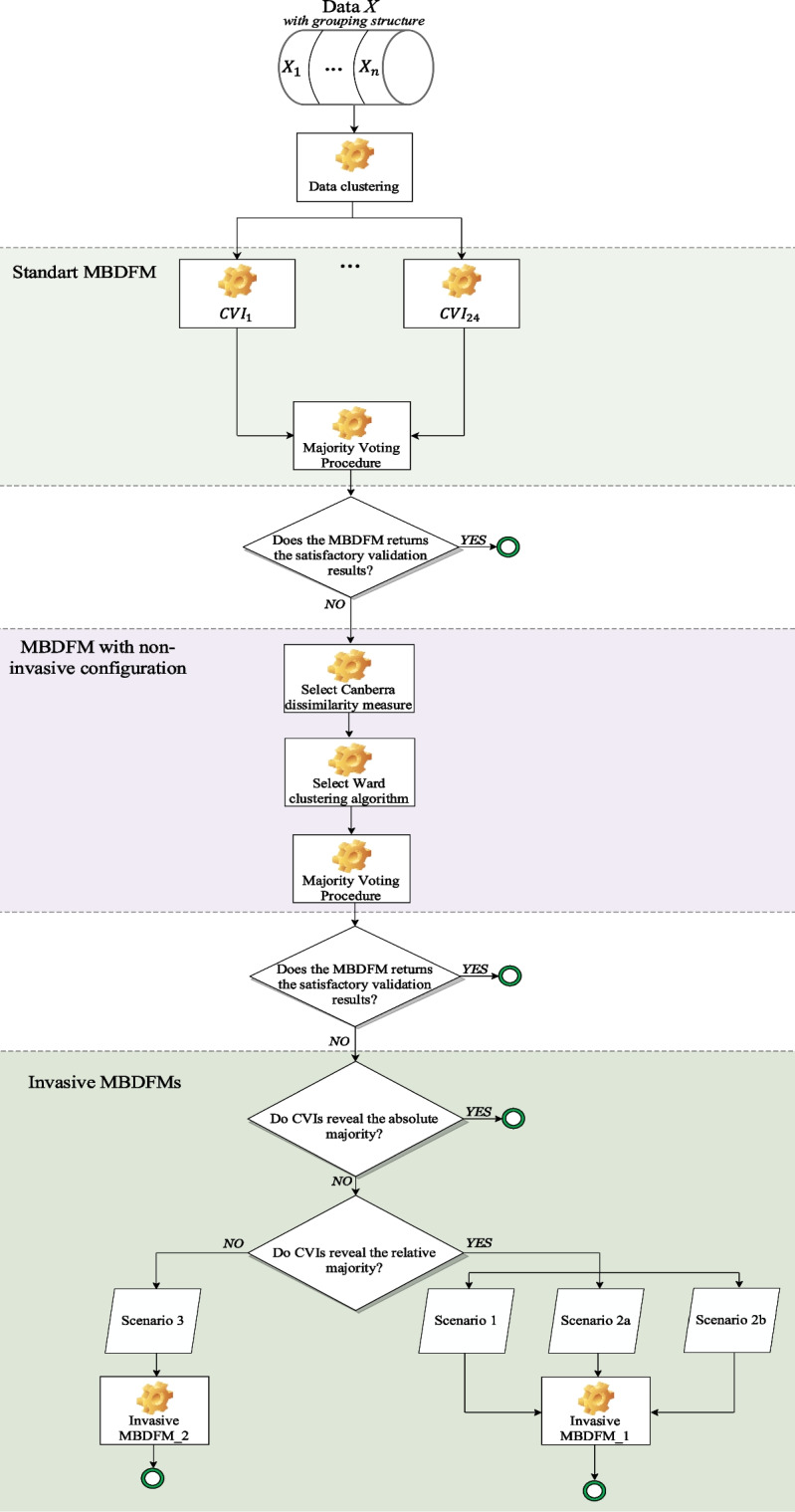
The block diagram of majority-based decision fusion methods. From top to bottom: standard MBDFM – method presented by Charrad et al. (2014); the MBDFM with non-invasive configuration and invasive MBDFMs are the methods created and implemented by Panskyi and Mosorov (2021)

In the literature on the clustering validation topics, one can find many alternative ways of building clustering validity indices, defining the combination strategy and estimation algorithm and justifying the final “true” number of clusters (Osamor & Osamor, 2020; Nerurkar et al., 2019; Kumar et al., 2019; Granichin et al., 2015; Santos & Embrechts, 2014). All these lead to a myriad of alternative simple and sophisticated clustering validation solutions. A variety of clustering validity indices are aimed at validating the results of clustering analysis and determining which clustering algorithm performs the best, for a particular experimental setup. However, comparative studies show that there is no optimal CVI which outperform the others (Dimitriadou et al., 2002; Milligan & Cooper, 1985) Moreover, there is no single way of establishing the quality of a partition by selecting the optimal CVI that is able to cope successfully with all the contexts under different conditions (Gurrutxaga et al., 2011; Hamalainen et al., 2017). Thus, the authors (Martinez-Tejada et al., 2022; Bhatia et al., 2020) remark that no single CVI is always the best or performs best on any given data set, dissimilarity measure or clustering algorithm. Some authors (Chisalita et al., 2020; Yang et al., 2017) created universal composite CVIs that combine multiple internal and external CVIs into one generalized index. Others, (Kryszczuk & Hurley, 2010; Yera et al., 2017) suggest using multi-criteria decision fusion clustering validation approaches, which are based on gathering all available CVIs and obtaining the final decision based on some predefined rule. This approach is more stable and robust since users avoid choosing a different CVI for each particular experimental environment. Moreover, the users don’t fully rely on the decision generated by the composite index, which in consequence could produce misleading validation results. Even the best composite CVI could make the mistake while producing the final validation decision. Having analyzed the most common clustering validation approaches the authors adopted, modified and enhanced the multi-criteria majority-based decision fusion method (MBDFM) (Charrad et al., 2014). The proposed MBDFMs are carefully described in previous work (Panskyi & Mosorov, 2021) with a detailed explanation of the experimental setup (clustering algorithms, dissimilarity measures, dimensionality, number of CVIs). The block diagram of the proposed MBDFMs is presented in Fig. 1. The MBDFMs require the initial data set to be clustered, therefore the pretest–posttest statistics should be collected.

The first step of data collection of students’ self-awareness towards the online security problems, was the opportunity for each student to see and analyze the questions. Three groups of students (before, during and after the pandemic) had the opportunity to see all questions (Q1–Q20) without answers. To collect the pretest statistics for each question (Q1–Q20), the students responded to a self-referring statement on a scale ranging from “1–25 = I think that I definitely don't know the correct answer”, "26–50 = I think that I might don't know the correct answer", "51–75 = I think that I might know the correct answer" to “76–100 = I think that I definitely know the correct answer”. Students, without the answer list preview, had to give a sincere and truthful assessment of how much they evaluate their knowledge and how much they are sure that they know the correct answer and are familiar with the topic of the question. They could choose any number falling within the range [1;100], whether integer or real. After the pretest statistics has been collected the list of answers to each survey’s questions (Q1-Q20) appeared immediately.

After each student answers the survey questions (Q1-Q20) there is an opportunity for each student to assess the correctness of their initial assumption. Thus, each student had the opportunity to see the correct answer to the question and confirm or refute their assumptions about the correctness of the chosen answer. To collect the posttest statistics for each question (Q1–Q20), the students responded to a self-referring statement on a scale ranging from “1–25 = I definitely didn't know the correct answer”, "26–50 = I assume that I didn't know the correct answer", "51–75 = I assume that I knew the correct answer" to “76–100 = I definitely knew the correct answer”. Students with the list of distractors and the correct answer had the opportunity to assess whether they thought correctly and to compare how close or far from the correct answer they were based on the range [1;100] of possible numbers. Thus, the posttest statistics had been collected.

**Fig. 2:**
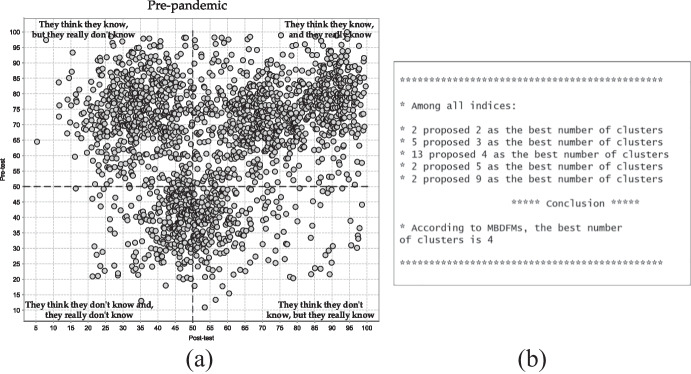
(**a**) Two-dimensional plot of pretest–posttest data set collected before the pandemic (**b**) The result of MBDFMs operation

After the initial data set has been created, the proposed MBDFMs have been applied. Additionally, to the initial data set, the MBDFMs require the user to input the range of a possible number of clusters, to perform clustering validation procedures. Since the survey consists of 20 questions, the range of possible clusters is [2…20]. The MBDFMs assume there always will be one cluster, and therefore the validation procedure starts with 2 clusters. The example of the MBDFMs applied to the pretest–posttest pre-pandemic data of students’ self-awareness of the Internet e-threats and perception of the online security has been shown in Fig. 2.

The scatter plot presented in Fig. 2 shows the distribution of the pretest–posttest data. The authors divided the scatter plot into four quarters: left bottom – students thought they didn't know the answer to the particular question and they really didn't know the answer, post-factum, after the correct answer appears; left top – students thought they knew the answer to the particular question but in fact, they were mistaken and didn't know the correct answer; right top – students assumed they knew the correct answer to the particular question and they really do; right bottom – students assumed they didn't know the correct answer, however, they made the right choice. The presented scatter plot illustrates the majority of data points located in the right top quarter then the top bottom and the least of data points are saturated in the bottom quarters.

The MBDFMs revealed four independent “true” clusters. The cluster notion in this analysis means a group of questions that may or may not be a problem for primary school students. Unfortunately, the MBDFMs only reveal and justify the “true” number of clusters, however, these validation methods don’t attribute the data point to each particular cluster. Moreover, the identified clusters may consist of answers to one or more questions, since pretest–posttest statistics were collected based on the whole set of questions of the survey. The overall picture illustrates the student's confidence towards the correct answer to a particular question, however, this confidence is reasonable only in the right top quarter. In the left top quarter, their confidence in their prior online security knowledge and self-awareness towards the Internet security problems had been translated into misleading and erroneous results. To summarize, the students before the pandemic show a higher self-assessment based on the high pretest–posttest score, which in turn would mean both, a justified level of reasonable confidence in their knowledge and learning outcomes or self-confidence and inflated self-esteem in overestimating their own potential of e-threats in the domain of online security.

**Fig. 3:**
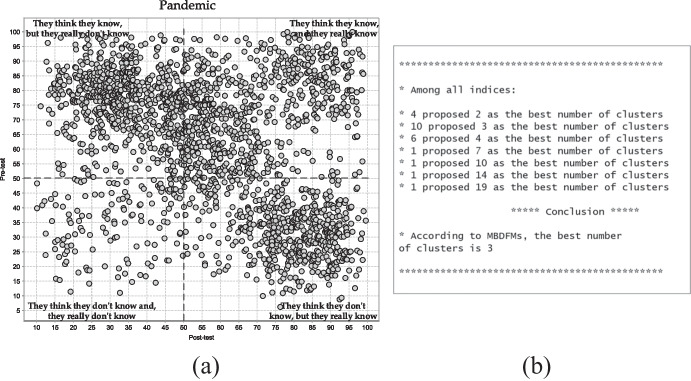
(**a**) Two-dimensional plot of pretest–posttest data set collected during the pandemic (**b**) The result of MBDFMs operation

The scatter plot presented in Fig. 3 shows the distribution of students’ responses during the pandemic challenging period. The majority of data objects fall into two top quarters and one bottom right quarter. Moreover, among all quarters the top left quarter gathered the biggest group of data objects. The situation differs significantly from those before the pandemic. The top left quarter corresponds to those students that assumed they know the correct answer however they gave the erroneous one. The explanation of this scenario requires a full and detailed analysis of the implementation procedures towards the distance education tasks. During the pandemic teachers did not pay appropriate attention to the cyberspace security issues, therefore the students’ responses fall into the top left quarter. Students were oversaturated with other school tasks and such necessary online security problems were not a priority at all. On the other hand, there is a fairly large group of data objects that are located in the bottom right quarter, which was not detected in students before the pandemic. Students revealed low self-awareness and confidence in the pretest procedure in this part of the scatter plot nevertheless, subconsciously, they knew the correct answer and therefore made the right decision in the posttest reevaluation.

The MBDFMs revealed three “true” separate clusters. The number of clusters decreased compared to the pre-pandemic period. Data object migration and the number of clusters reduction are directly related to the enforcement measures to fight the pandemic at the national level, for the protection of, support for, and assistance to primary school students. The measures were rather temporary, impermanent and illusory, isolated procedures than complex dynamic structures, that should perform educational tasks in the field of online security through a set of activities, actions, and organisational undertakings. The provided measures during the pandemic did not express in sufficiently precise terms the necessity of continuing the adaptation of the schools' education Internet-related security environment as well as e-threats protection strategies with proper ICT infrastructure and student awareness towards the online dangers, primarily they were focused, directed and intended to ensure appropriate crisis-prompted distance education and learning conditions.

**Fig. 4:**
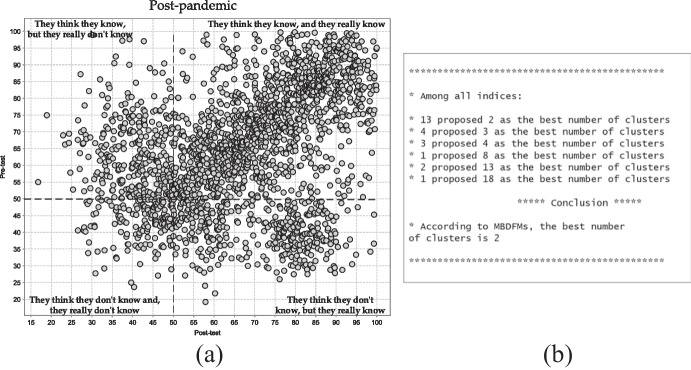
(**a**) Two-dimensional plot of pretest–posttest data set collected after the pandemic (**b**) The result of MBDFMs operation

The scatter plot presented in Fig. 4 presents the distribution of students’ responses during at present time, at the post-pandemic period. The majority of all data objects are located in the top right quarter. The students assumed they knew the correct answer and in fact, they really did. This means that in post-pandemic time, Polish primary school students have high self-awareness and perception of online security problems. The measures applied during the pandemic nowadays have contributed to the raising of the standards and quality of educational security ICT infrastructure, moreover, they have boosted the productiveness of actions and the efficiency of the schools toward the online security policy. The consequence of those changes, which are becoming more and more evident, is the significant increment in students' digital and Internet security competencies. At present, authorities perform permanent supervision and concurrent control by means of guidelines and recommendations over the activities of schools on ensuring the protection of cyberspace. Moreover, teachers, using new approaches, practices, measures and procedures pay much more attention to Internet security and personal information protection.

The MBDFMs revealed two separate individual clusters. During post-pandemic time, the number of clusters decreased compared to the pandemic time. Only now has it been noticed how significant the impact of the technical and organisational measures taken. The ideal situation would be the presence of one cluster general cluster in the right top quarter. In such a scenario, students' self-awareness towards online security problems would fully correspond to their confidence in the acquired knowledge and skills. Nevertheless, the positive tendency consists of properly identifying, recommending and literate distributing measures which contribute to better performance by all entities involved in the coordinative situation in the future (authorities, schools, teachers, parents and primary school students). In the light of the changes in education, redefining the schools' activities involving the prevention, explanation and analysis of online security problems seems to be inevitable. Ensuring adequate protection is now impossible without engaging primary school students. Nowadays, special attention should be drawn to building an efficient instrument for the prompt making of correct key decisions. Moreover, cyberspace threats and dangers should be clearly explained, interpreted and visualised to every student in primary school settings. The more time spends on the educational measures toward the online security, the less likely the primary school students to fall into the cyberspace traps.

## Conclusions

The majority of Polish society lives in the world of digital content and services, permeating everyday life like no technology in the past. Therefore, Polish schools must fully, substantively and safely operate in the digital environment, using educational resources available online: multimedia content, applications, platforms and associated interactive teaching methods. Fully—that is, not selectively, but consistently across all subjects; substantively—that is, understanding the specifics of online digital resources and tools and their methodological applications, as well as safe—and therefore being aware of e-threats and knowing how to react to them. The most important for ensuring the foundations of digital security at school are preventive measures carried out against and with the participation of all entities of the school community: students and their parents, principals, teachers and other school employees (e.g. psychologists, educators, secretarial staff). These measures should be systemic, continuous, long-term and coordinated, and their scope should be included in the educational and preventive program implemented at the school.

With respect to the first research question, the pandemic had a significant influence on Polish primary school students' educational learning outcomes in information and Internet security. However, it should be emphasized that this impact was significant only in a few selected questions. In general, so far, Polish primary school students have not fully felt the pandemics' influence on learning outcomes regarding the online security problems. The educational crisis engendered by the COVID- 19 pandemic was the perfect litmus test for authorities, schools, teachers, and students to implement and coordinate wide, versatile, and interdisciplinary educational, organisational and technical measures. As a result, some schools implement a Digital Safety Plan in the form of a contract, agreed upon and concluded between all co-creators of the educational environment: students and their parents as well as teachers and other school employees. Such a contract—with a well-balanced set of rights and obligations of all signatories—stimulates in students a sense of shared responsibility for the situation at school and builds a sense of subjectivity as an adult partner in school life.

With respect to schools, they introduce the annual minimum scope of prevention measures that include: meetings of the entire school community, meetings of the school community with experts, organisation of a school digital safety day, competitions and contests organisation—based on the rivalry between classes—on Internet security, extracurricular activities, educational projects considering new ICTs, etc. The subject of online security, in some schools, has already appeared on the school's website and the school's profiles on social networks as a separate issue.

With respect to teachers, they participate in different pieces of training, courses and workshops on selected issues of online security and e-threats, using funds at the disposal of the school management to raise the qualifications of teachers or funds from external projects (e.g. EU, curatorial offices, Ministry of National Education). In their daily didactic work, many teachers strive to include Internet security issues in teaching non-informatics subjects, to a greater extent including them in STEM subjects.

Responsibility for primary school students' education in the field of online security lies with both parents and teachers, especially in informatics and related subjects. Not all parents are aware of the dangers lurking online, and in some cases, the parents' awareness in this regard is even lower than that of their children. For this reason, it is primarily the informatics-educated teachers who should support young people in acquiring knowledge about cyberspace in general and online security in particular. At present, parents receive basic educational support, where the cooperation of the school with parents in the first place consists in making them aware of the importance of their role in preparing their children for the secure use of the Internet.

When investing in the school's digital ICT infrastructure, one should strive to purchase ICT devices adapted to the needs and specificity of their use by students and the professional development of the school's Internet network system. Online security is strongly correlated with the quality of the ICT school infrastructure. It is also favored by the use of external educational platforms and cloud solutions. In addition to tackling e-threats at a technical level, schools nowadays install updated traffic-blocking systems to filter out inappropriate, undesirable and illegal content for students. The technical cyberspace security of schools should be the responsibility of specialists. It is necessary to hire a person professionally responsible for the schools' ICT infrastructure. Moreover, this person should not combine their tasks with the role of an informatics or STEM teacher. Its responsibilities must mainly include ensuring the reliability and safety of ICT equipment and networks so that teachers and students can use them without wasting time on adjustments, repairs and installations.

Systematic preventive measures at school significantly reduce the scope of e-threats in cyberspace, but are not able to completely eliminate them. In the case of a security e-threat incident, school measures should be characterized by: openness, quick identification of the problem—determining harmful or illegal behavior—and its solution adequate to the level of risk it caused at school. Similarly—without undue delay, substantively—using the knowledge of experts and good practices from other institutions, the school should react in the case of appeared problems. It is worth emphasizing that there is no "golden prescription" that can be used in all cases of e-threats. Principals and teachers must consider the context of individual cases as well as their school and environmental background in order to respond appropriately with an adequate level of responsibility.

Current research has proved that data clustering techniques can be used to enrich decision making in different domains such as finance, healthcare, statistics and biology by transforming raw data into crucial information. Educational data clustering is also important in analyzing data to improve pedagogical aspects of teaching and learning. In recent years, the biggest challenge that educational institutions facing is the explosive growth of educational data. These massive amounts of data stored need some data clustering techniques to retrieve useful and meaningful information from the data sets. Data clustering helps to classify students in a well-defined cluster to find the behavior of their learning styles. Moreover, clustering as a preprocessing step helps to predict students’ performance in different educational environments.

With respect to the second research question, the presented MBDFMs illustrate a significant impact of the pandemic period on Polish primary school students' self-awareness and perception of information and online security. The findings from this study imply that MBDFMs offer great opportunities for researchers to use data collected from student-oriented surveys to explore the intrinsic structures, learning outcomes or behaviors. The results offer insights into two areas: first, MBDFMs can be successfully used to identify students' self-awareness and perception in different educational domains. Second, the MDBFMs provide a means of understanding the cyberspace security problems so as to develop guidance, measures and support aligned to students’ needs hence offering the opportunity for teachers and parents to provide targeted interventions for each of the formed cluster groups. The results from this study also contribute to the evaluation of MBDFMs by giving insights into how to build machine learning predicting models that can be used by each particular school to provide future measures and organisational actions. Finally, the authors believe in the potential possibility of combining MBDFMs, clustering and classification procedures for further adoption in Poland's educational system for real-time data analysis. The additional work in the clustering validation area could include aspects such as: testing the new multi-criteria decision fusion approaches proposed over a large number of real data sets and designing new fusion strategies using other novel CVIs, moreover, complex data structures and heavy overlapping clusters would appear to be the most interesting and promising factors to analyze in greater depth.

The subject of online security is insufficiently known by primary school students. Taking into account the revealed results, the authors believe that teaching programs in the field of online security should be revised and complemented by the additional classes devoted to this subject, with particular emphasis on active teaching methods based on case studies, flipped education, problem-based and game-based learning. These modern teaching methods will show students the existing online security problems in an interesting, interactive and creative way.

## Data Availability

Derived data supporting the findings of this study are available from the corresponding author on request.

## Appendix 1

Table 3

**Table 3:** The survey provided to the primary school students regarding the Internet and information security as well as e-threats and online dangers

Short name	Questions	Answers
Q1	Viruses are usually attached to files with the extension:	○.*exe and.com* ○.gif ○.pdf ○.jpg and.bmp
Q2	What can a virus do with your ICT device? Choose the best answer	○ May cause freezing of the operating system ○ It can damage the operating system ○ It can send fake messages from your ICT device or to your ICT device ○ *All the above answers are correct*
Q3	How can a virus get into your ICT device? Choose the best answer	○ By downloading the program from a random website ○ Via USB memory or other external drives ○ By opening an infected email attachment ○ *All the above answers are correct*
Q4	Which of the programs protects your ICT device against e-threats	○ *Antivirus program* ○ Anti-spyware program ○ Disk defragmenter ○ None of the above
Q5	What is an antivirus program?	○ A program that removes all data from the ICT device ○ A program that opens viruses access to your ICT device ○ *A program that blocks and removes Internet viruses* ○ A program that isolates the ICT device from the Internet
Q6	Why are some websites considered to be unsafe?	○ All websites are safe ○ They can attack our ICT device ○ They have too many displayed ads ○ *They may contain viruses that will spread to our ICT device*
Q7	How many antivirus programs should I have on my computer?	○ The more the better ○ Two. One to fight viruses and the other to fight worms and rootkits ○ *One, because more of them can fight each other* ○ We can choose not to install any—just not open websites
Q8	Antivirus by connecting via the Internet to the database of the latest virus definitions:	○ *performs update* ○ performs refresh ○ checks the license ○ cleans your ICT device of viruses
Q9	The addresses of websites that allow you to log in safely and enter passwords begin with:	○ http: // ○ ftp: // ○ doi: // ○ *https: //*
Q10	You have received an e-mail: "You won the computer. You will receive it when you enter your account password"	○ You send an e-mail with your password immediately ○ You write back that you can send it by regular mail because e-mail is dangerous ○ You provide your data and password so that it is known to whom to send the computer ○ *You are not sending your password because someone might misuse your account for a criminal purpose*
Q11	Should you provide your personal data on the Internet, together with your address and telephone number?	○ *Never, because someone might take advantage of them and you could be in trouble* ○ Yes, then everyone gets to know you better ○ Yes, you will have a better chance of finding new friends ○ Yes, because everyone will be able to write or call you
Q12	What information about yourself can you safely provide on the web?	○ Your home address ○ Your phone number ○ *Your interests* ○ Your first and last name
Q13	Which nickname will be safe to use during chat conversations?	○ jula_12 ○ tom2001 ○ ola_kowalska ○ *cloud*
Q14	In which case is it safe to provide your real personal data online?	○ On the page where you can check your horoscope ○ *When you perform online shopping on a trusted website* ○ By joining the discussion on the forum ○ By placing them on the website created by yourself
Q15	Someone bothers you on the Internet. What are you doing?	○ *You do not write back and notify your parents about it or report it to Helpline.org.pl* ○ You write him/her back to write to you at another time because you are busy ○ You scare him/her with your older brother, strong friend or father ○ You write back to him/her to get off your back
Q16	Can you, with absolute certainty, find out the age of the person you are chatting with on the Internet?	○ *This is not possible* ○ Yes, after I read the information in his/her profile ○ Yes, by IP number. Yes, I can ask his/her ○ There is always some way to find it out
Q17	In the network, someone with an unknown login asks you to send your photo to him/her. What are you doing?	○ You choose the most beautiful photo and send it immediately ○ *You don't send it and tell your parents about it* ○ You answer that you can send your photo, but only by traditional mail ○ You correct your photo in a graphics program and send it
Q18	You have a nice conversation with your friend on the Internet. He says his name is Tomek and he is 14 years old	○ He's who he says, for sure. He's nice so he can't lie ○ *You can't be sure that he is the person that he says* ○ He sent you a photo, so he is definitely not lying ○ He said that he knows your parents and your friends, so he is definitely not lying
Q19	A person you meet on the Internet offers you a meeting. What are you doing?	○ *You make an appointment in a public place and you go to a meeting with your friends* ○ You are willing to go for a walk in the forest because you want to get to know him/her also in the real world ○ You had a good time talking on the web. You go to a meeting without telling anyone ○ You go to a meeting, and you tell your parents that you are going to school for additional sports activities
Q20	To take care of your online safety, you should? Choose the best answer	○ Keep your online profiles private ○ Do not write down your passwords in places where someone can see them ○ Secure your computer with an appropriate anti-virus program ○ *All the above answers*

The correct answer is typed in *Italic*
